# The Liver-Selective Thyromimetic T-0681 Influences Reverse Cholesterol Transport and Atherosclerosis Development in Mice

**DOI:** 10.1371/journal.pone.0008722

**Published:** 2010-01-15

**Authors:** Ivan Tancevski, Egon Demetz, Philipp Eller, Kristina Duwensee, Julia Hoefer, Christiane Heim, Ursula Stanzl, Andreas Wehinger, Kristina Auer, Regina Karer, Julia Huber, Wilfried Schgoer, Miranda Van Eck, Jonathan Vanhoutte, Catherine Fievet, Frans Stellaard, Mats Rudling, Josef R. Patsch, Andreas Ritsch

**Affiliations:** 1 Department of Internal Medicine, Innsbruck Medical University, Innsbruck, Austria; 2 Division of Biopharmaceutics, Leiden/Amsterdam Center for Drug Research, Gorlaeus Laboratories, Leiden University, Leiden, The Netherlands; 3 Département d'Athérosclérose, Institut Pasteur de Lille, Lille, France; 4 Laboratory of Pediatrics, Center for Liver, Digestive and Metabolic Diseases, University Medical Center Groningen, Groningen, The Netherlands; 5 Molecular Nutrition Unit, Departments of Medicine and Biosciences and Nutrition, Karolinska Institute at Center for Endocrinology, Metabolism and Diabetes, Karolinska University Hospital, Stockholm, Sweden; Leiden University Medical Center, Netherlands

## Abstract

**Background:**

Liver-selective thyromimetics have been reported to efficiently reduce plasma cholesterol through the hepatic induction of both, the low-density lipoprotein receptor (LDLr) and the high-density lipoprotein (HDL) receptor; the scavenger receptor class B type I (SR-BI). Here, we investigated the effect of the thyromimetic T-0681 on reverse cholesterol transport (RCT) and atherosclerosis, and studied the underlying mechanisms using different mouse models, including mice lacking LDLr, SR-BI, and apoE, as well as CETP transgenic mice.

**Methodology/Principal Findings:**

T-0681 treatment promoted bile acid production and biliary sterol secretion consistently in the majority of the studied mouse models, which was associated with a marked reduction of plasma cholesterol. Using an assay of macrophage RCT in mice, we found T-0681 to significantly increase fecal excretion of macrophage-derived neutral and acidic sterols. No positive effect on RCT was found in CETP transgenic mice, most likely due to the observed decrease in plasma CETP mass. Studies in SR-BI KO and LDLr KO mice suggested hepatic LDLr to be necessary for the action of T-0681 on lipid metabolism, as the compound did not have any influence on plasma cholesterol levels in mice lacking this receptor. Finally, prolonged treatment with T-0681 reduced the development of atherosclerosis by 60% in apoE KOs on Western type diet. In contrast, at an earlier time-point T-0681 slightly increased small fatty streak lesions, in part due to an impaired macrophage cholesterol efflux capacity, when compared to controls.

**Conclusions/Significance:**

The present results show that liver-selective thyromimetics can promote RCT and that such compounds may protect from atherosclerosis partly through induction of bile acid metabolism and biliary sterol secretion. On-going clinical trials will show whether selective thyromimetics do prevent atherosclerosis also in humans.

## Introduction

It has been known since 1930 that hyperthyroidism is associated with reduced plasma cholesterol levels [1], [reviewed in 2], and since then many efforts were made to exploit the ability of thyroid hormones (TH) to lower cholesterol. In the late 1960s, a large clinical trial of dextrothyroxine (D-T_4_) therapy was conducted, as part of The Coronary Drug Project by the National Institutes of Health, which aimed to answer the question whether cholesterol reduction may prevent coronary heart disease [3]. However, the unfavorable recruitment of patients together with the accidental employment of preparations contaminated with the enantiomer of D-T_4_ resulted in a higher proportion of deaths in the D-T_4_ treated group, leading to the discontinuation of clinical studies with TH analogs in the 1970s [4], [5]. With the introduction into clinical practice of HMG-CoA reductase inhibitors, usually known as ‘statins’, to lower plasma cholesterol in the mid 1980s, efforts on the development of TH analogs slowed. However, the last 20 years saw the development of thyromimetic compounds selective for the liver and/or the β1-isoform of the TH receptor which all were shown to efficiently lower plasma cholesterol without concomitant deleterious effects on the heart [4], [5]. Several selective thyromimetics have been shown to be useful lipid-lowering compounds in animal studies [6], [7], [8] resulting in clinical trials [9].

At present, it is believed that thyromimetics constitute useful lipid-lowering therapeutic agents as they lead to a marked reduction of low-density lipoprotein LDL cholesterol (LDL-C) by enhancing the hepatic expression of the LDL receptor (LDLr) [4], [5]. Recently, it has been shown that liver-selective thyromimetics upregulate hepatic SR-BI, which is an important component in reverse cholesterol transport (RCT) [7], [10]. By their dual action on LDL metabolism and RCT, thyromimetics could be expected to counteract atherosclerosis.

In the present study, we investigated the effect of the liver-selective thyromimetic T-0681 on RCT by measuring the transport of cholesterol from macrophages to feces. We further studied the impact of T-0681 on the development of atherosclerosis in mice, and analyzed the underlying mechanisms.

## Results

### Effect of the Thyromimetic T-0681 on Lipid Metabolism in Wild-Type Mice

In preliminary dose-titration studies in wild-type (WT) mice we observed a marked increase of hepatic SR-BI expression at 36 nmol/kg/d T-0681, and a concomitant 50% decrease of plasma cholesterol. Higher doses than 36 nmol/kg/d showed no further lipid-lowering effect (data not shown). Accordingly, Parini and coworkers recently presented data on SR-BI-inducing properties of the thyromimetic GC-1 in liver of WT mice [7].

**Figure 1:**
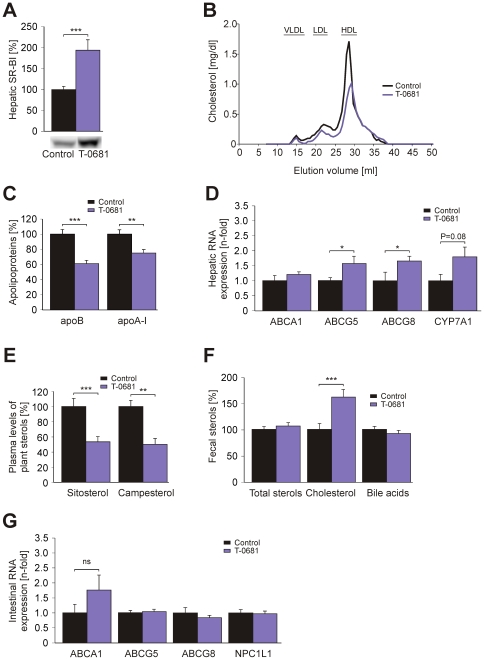
The liver-selective thyromimetic T-0681 promotes reverse cholesterol transport. Chow-fed WT mice were treated with T-0681 (36 nmol/kg/d) or PBS. (A) Western blot showing hepatic expression of SR-BI (N = 6). (B) FPLC analysis of pooled plasma from control and T-0681-treated mice (N = 6). (C) Plasma concentrations of apoB and apoA-I (N = 6). (D) Taqman real-time PCR analysis of hepatic ABCA1, ABCG5, ABCG8, and CYP7A1 (N = 4–10). (E) Analysis of fecal sterols (N = 6), and (F), plasma levels of diet-derived phytosterols, normalized to cholesterol (N = 5). (G) Taqman real-time PCR analysis of intestinal cholesterol transporters ABCA1, ABCG5, ABCG8, and NPC1L1 (n = 4–10). *P<0.05, **P<0.01, ***P<0.001 versus corresponding controls; ns, non significant; data presented in % are normalized to the respective controls.

In subsequent experiments in WT mice, 36 nmol/kg/d of T-0681 was found to reproducibly increase hepatic SR-BI expression and to decrease both LDL-C and high-density lipoprotein cholesterol (HDL-C, **Figure 1A, B**). This effect was paralleled by decreased plasma contents of apoB and apoA-I (**Figure 1C**). Real-Time PCR measurements of liver specimens revealed increased mRNA levels of CYP7A1, the rate-limiting enzyme for conversion of cholesterol into bile acids, and an increased expression of hepatic ABCG5 and ABCG8, which are known to promote biliary sterol secretion upon dimerization (ABCG5/G8, **Figure 1D**) [11]. T-0681 treatment was associated with increased fecal neutral sterol loss and strongly reduced phytosterol levels in plasma (−50%), which are known to reflect intestinal cholesterol absorption (**Figure 1E–F**) [12].

No significant changes of the intestinal cholesterol transporters ABCA1, ABCG5/G8 and Niemann-Pick C1 Like 1 [13], [14] were found (**Figure 1G**), no differences in food intake were observed (data not shown).

### Reverse Cholesterol Transport

**Figure 2:**
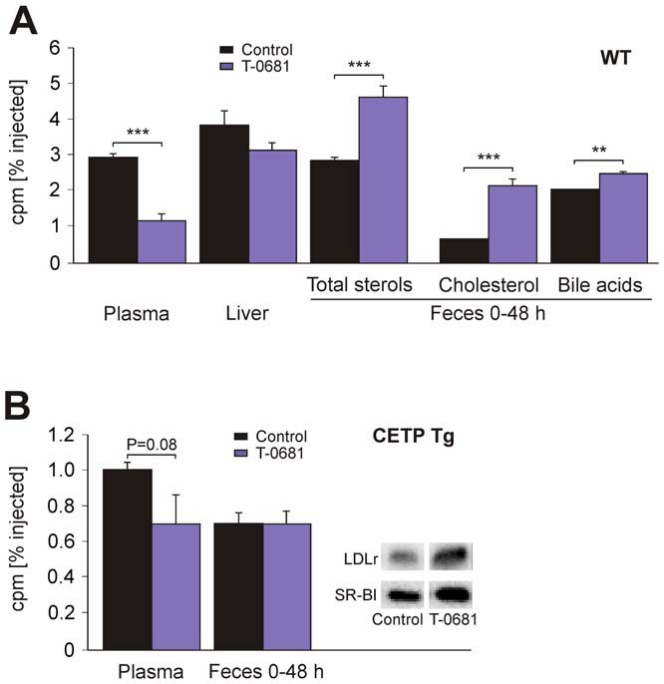
Influence of T-0681 on macrophage reverse cholesterol transport *in vivo*. (A) WT mice were treated with T-0681 (36 nmol/kg/d) or PBS, and the treatment was continued during the 48-hours RCT study. 12 days into treatment, cholesterol-loaded, [^3^H]-labeled J774 macrophages were injected intraperitoneally, and 48 hours later tracer was measured in plasma, liver, and fecal sterols (N = 10). (B) Macrophage *in vivo* RCT in CETP Tg mice was performed as described in (A) (N = 5); inset: Western blot showing hepatic expression of LDLr and SR-BI; each lane represents pooled protein extracts from 5 mice per group. **P<0.01, ***P<0.001 versus corresponding control.

Recently, Rader and coworkers developed the first *in vivo* method to trace RCT specifically from macrophages to feces [15]. After intraperitoneal injection of [^3^H]-cholesterol-labeled macrophages, the tracer is measured in plasma, liver, and feces. In our experiments, T-0681 treated WT mice showed a strong decrease of plasma [^3^H]-cholesterol levels and no changes in hepatic tracer content. Most importantly, fecal excretion of neutral and acidic sterols was significantly increased in T-0681-treated animals (**Figure 2A**). Similar effects were observed in macrophage RCT studies using primary murine bone marrow-derived macrophages (data not shown). Our data resemble the results from macrophage RCT studies in SR-BI overexpressing animals [16]. In addition to increased hepatic SR-BI expression, our mice also exhibited increased hepatobiliary secretion of sterols via ABCG5/G8 and CYP7A1, which may substantially contribute to the promotion of RCT by T-0681.

RCT in humans is different from that found in rodents in that cholesterol from the periphery can be transported to the liver either directly via HDL particles or, after transfer to VLDL and LDL mediated by the cholesteryl ester transfer protein (CETP), via apoB-containing lipoproteins [17]. We therefore tested whether treatment with a thyromimetic compound would also promote RCT in an animal expressing CETP, and performed macrophage RCT experiments in CETP transgenic mice (CETP Tg), overexpressing human CETP under the control of its own promoter. In CETP Tg mice, treatment with T-0681 significantly reduced plasma cholesterol (82±4 mg/dL vs. 56±2 mg/dL, control vs. T-0681, *P*<0.001). Plasma [^3^H]-cholesterol levels tended to be reduced (**Figure 2B**). However, there was no significant increase in fecal [^3^H]-sterol levels, although both SR-BI as well as LDLrs were found increased in livers of T-0681 treated animals (SR-BI 1.35-fold of controls, *P* = 0.07; LDLr 1.8-fold of controls, *P*<0.01; **Figure 2B**, inset). The appropriate delivery of [^3^H]-cholesterol to the liver may have been hampered by reduced cholesteryl ester transfer from HDL to LDL/VLDL particles, as suggested by reduced plasma CETP mass (1.74±0.12 vs. 1.38±0.12, control vs. T-0681, *P* = 0.07). In addition, in contrast to WT mice, no induction of hepatic CYP7A1 and ABCG5/G8 was observed (summarized in Table 1), which may have slowed down the transport of [^3^H]-sterols from the liver to the bile.

**Table 1:** Hepatic regulation of SR-BI, LDLr, ABCG5/G8, CYP7A1 by T-0681.

Mouse model	SR-BI	LDLr	ABCG5/G8	CYP7A1
**WT**	↑	−	↑	↑
**SR-BI KO**	KO	↑	↑↑	↑↑↑
**LDLr KO**	−	KO	↑↑	↑↑
**CETP Tg**	↑	↑	−	−
**apoE KO 4-wk**	−	−	−	−
**apoE KO 8-wk**	−	−	↑↑	↑↑

The table summarizes the relative hepatic expression of the analyzed proteins derived from all the experiments presented in this study. WT, wild-type mice; KO, knock-out mice; 4-wk, 4 weeks study; 8-wk, 8 weeks study; −, no change; ↑, 1.5–2 - fold of control; ↑↑, more than 2-fold of control; ↑↑↑, more than 4-fold of control.

### Relative Contribution of SR-BI and LDLr to the Effect of T-0681 on Lipid Metabolism

Interestingly, we observed no effect of T-0681 on hepatic LDLr expression of WT mice (data not shown). This finding is in agreement with data by Parini and coworkers [7], but contrasts the view of thyromimetics to act via induction of LDLr expression [18].

**Figure 3:**
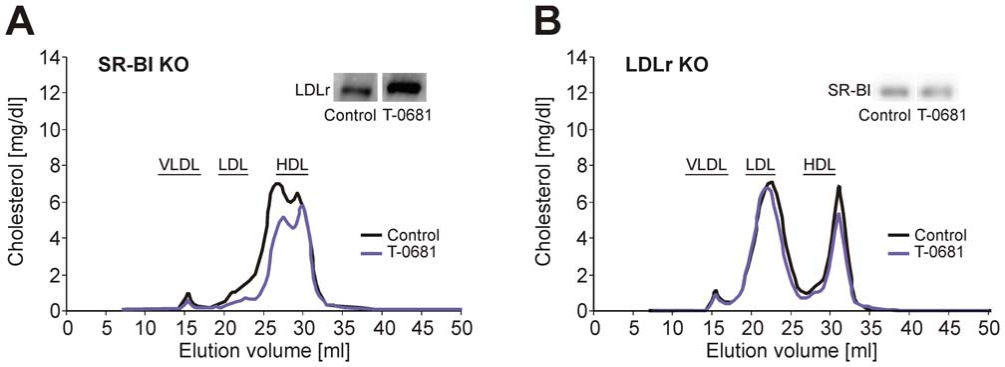
Relative contribution of SR-BI and LDLr to the cholesterol-lowering action of T-0681. (A) FPLC analysis of pooled plasma from SR-BI KO mice with and without T-0681 treatment (36 nmol/kg/d; N = 4); inset: Western blot showing hepatic expression of LDLr; each lane represents pooled protein extracts from 4 mice per group. (B) FPLC analysis of pooled plasma from LDLr KO mice with and without T-0681 treatment (36 nmol/kg/d; N = 4); inset: Western blot showing hepatic expression of SR-BI; each lane represents pooled protein extracts from 4 mice per group.

Interestingly, we found a marked increase in LDLr expression in the liver of CETP-expressing animals treated with T-0681, including the here presented CETP Tg mice (**Figure 2B**, inset), as well as in the recently studied hypercholesterolemic New Zealand White (NZW) rabbits [10], which naturally express plasma CETP. Moreover, T-0681 significantly increased hepatic LDLrs in SR-BI KO mice (2-fold of controls, *P*<0.01), along with a marked decrease in plasma cholesterol (**Figure 3A**). In contrast, T-0681 did not affect plasma cholesterol in LDLr KO mice nor did it induce the hepatic expression of SR-BI (**Figure 3B**). Regulation of hepatic CYP7A1 and ABCG5/G8 is summarized in Table 1.

### The Dual Role of a Liver-Selective Thyromimetic on Atherosclerosis

**Figure 4:**
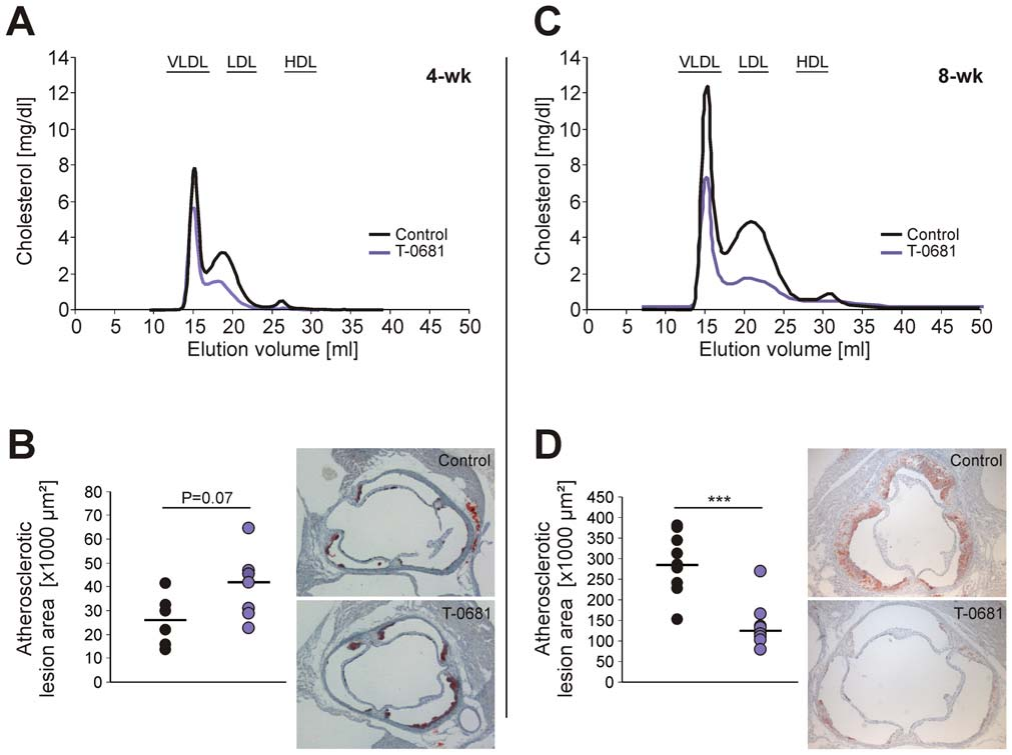
Influence of T-0681 on atherosclerosis development in apoE KO mice. Western type diet-fed apoE KOs were treated with T-0681 (36 nmol/kg/d) or PBS for 4 weeks (A, B) (N = 7) and 8 weeks (C, D) (N = 10). (A, C) FPLC analysis of pooled plasma from control and T-0681-treated mice. (B, D) Statistical comparison of the atherosclerotic lesion size in histological sections from the aortic root. ***P<0.001 versus corresponding control.

To study the impact of a selective thyromimetic on the development of early atherosclerosis, apoE KO mice were fed a Western type diet for 4 weeks and were concomitantly treated with 36 nmol/kg/d T-0681 or a placebo control. At study termination, T-0681 treated animals showed a slight increase in mean atherosclerotic lesion area on the one hand, and a decrease of cholesterol in apoB-containing lipoproteins on the other hand (**Figure 4A, B**); liver cholesterol was unaffected (11±1 mg/g liver vs. 12±1 mg/g liver, control vs. T-0681, *P* = 0.44). No difference was observed in the hepatic expression of SR-BI, LDLr, ABCG5/G8 and CYP7A1 (summarized in Table 1).

Prolonged treatment with T-0681 for 8 weeks strongly inhibited the progression of atherosclerotic lesions in apoE KOs, as shown in **Figure 4C, D**. The decrease in total plasma cholesterol was more pronounced, when compared to the 4-weeks study, and lipid accumulation in the liver was clearly inhibited by T-0681 treatment (26±2 mg cholesterol/g liver vs. 13±2 mg cholesterol/g liver, control vs. T-0681, *P*<0.001). There was no difference in both hepatic SR-BI and LDLr protein expression. However, we found a 3-fold upregulation of ABCG5, and a 2-fold increase of CYP7A1 in livers of T-0681 treated apoE KOs (*P*<0.001 for both, summarized in Table 1).

### Influence of T-0681 on Macrophage SR-BI Expression and on Plasma Cholesterol Efflux Capacity

**Figure 5:**
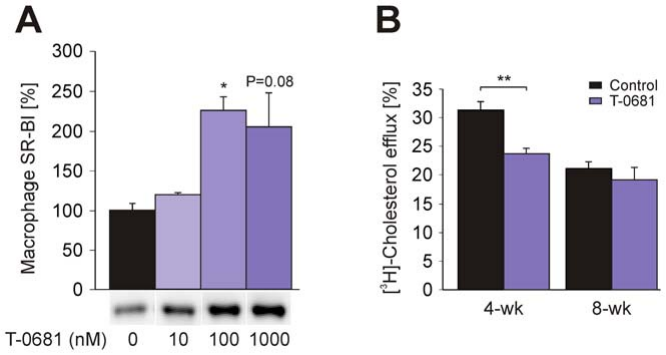
T-0681 increases macrophage SR-BI expression and affects macrophage cholesterol efflux. (A) Western blot showing expression of SR-BI in J774 macrophages treated with T-0681 at the indicated concentrations for 24 h (N = 3). (B) [^3^H]-cholesterol labeled J774 macrophages were treated with vehicle or 100nM T-0681 for 24 h, after which they were incubated with 2.5% pooled mouse serum from the 4-weeks and the 8-weeks study. Bars indicate mean [^3^H]-cholesterol efflux from triplicates. *P<0.05, **P<0.01 versus corresponding control.

To further understand the increase of small fatty streak lesions at 4 weeks in apoE KO mice displaying reduced lipid levels, we considered the analysis of the expression of cholesterol transporters in macrophages, and tested the plasma efflux capacity of mice from the 4-weeks and the 8-weeks study. As shown in **Figure 5A**, we found a dose-dependent increase of the SR-BI protein expression in macrophages treated with T-0681, and no effect of the thyromimetic on ABCA1 protein expression (not shown). SR-BI is known to mediate either cholesterol uptake or efflux, depending on the transmembrane cholesterol gradient [19], [20]. In our experiments, cholesterol efflux was significantly decreased in macrophages treated with T-0681 and incubated with the respective plasma from the 4-weeks study, compared to controls (**Figure 5B**). Interestingly, there was no difference in cholesterol efflux between controls and T-0681, when macrophages were incubated with the respective plasma from the 8-weeks study (**Figure 5B**). These findings suggest that, probably due to an unfavorable plasma lipoprotein composition, macrophages within the arterial wall may have been overloaded with cholesterol via SR-BI.

## Discussion

We previously showed that prolonged treatment with the liver-selective thyromimetic T-0681 dramatically reduced the atherosclerotic lesion area in NZW rabbits [10]. Using another animal model of dyslipemia, namely the apoE KO mouse, here again we show that prolonged treatment with T-0681 reduces late atherosclerosis development, which was associated with a decrease in the circulating levels of pro-atherogenic apoB-containing lipoproteins and increased hepatic expression of ABCG5/G8 and CYP7A1. Previously, ABCG5/G8 transgenic mice were reported to display enhanced biliary cholesterol secretion, increased neutral sterol loss via the feces, and strongly reduced absorption of dietary sterols [11]. Interestingly, T-0681 treated mice with increased ABCG5/G8 expression exhibited the same characteristics. However, liver-specific overexpression of ABCG5/G8 alone did not protect from atherosclerosis development [21]. T-0681 treated mice also showed enhanced conversion of macrophage-derived cholesterol into bile acids via CYP7A1, which constitutes a major pathway for cholesterol elimination. Moreover, hepatic overexpression of CYP7A1 was previously shown to significantly reduce plasma LDL-C levels [22]. In T-0681 treated mice, hepatic LDLr seems to be essential for functional bile acid synthesis as well as for biliary sterol secretion, as we and others showed that thyromimetics do not affect plasma cholesterol in LDLr KO mice [8].

Interestingly, after only 4 weeks of Western-type diet feeding, T-0681 slightly increased the development of small fatty streak lesions in apoE KO mice. There was a moderate decrease in plasma cholesterol levels, and no change in hepatic LDLr, SR-BI, ABCG5/G8 and CYP7A1 expression. In contrast to the 8-weeks study, there was no reduction in hepatic cholesterol content in T-0681 treated animals, which may have inhibited the induction of the studied cholesterol receptors/transporters [10]. At the moment, it remains elusive which mechanisms cause the decrease in plasma cholesterol at the early 4-weeks time-point. Further experiments are needed to elucidate the enterohepatic circulation of bile acids as well as the absorption of dietary sterols in the hyperlipidemic apoE KO model in more detail.

We further tried to understand the moderate increase in small fatty streaks upon thyromimetic treatment observed in the 4-weeks study. One possible explanation may be the fact that T-0681 was found to increase SR-BI in macrophages, where this receptor was shown to exert a dual role in atherosclerosis development. It is known that SR-BI mediates cholesterol flux across the plasma membrane, depending on the transmembrane cholesterol gradient [19], [20]. As suggested by the study of Van Eck and colleagues [23], at an early stage of atherosclerosis, higher levels of SR-BI may have promoted the (re-) uptake of cholesterol into macrophages. In contrast, at 8 weeks of treatment, where markedly reduced levels of cholesterol were found in the plasma of T-0681 treated mice, increased macrophage SR-BI levels may not have affected the efflux of cholesterol to HDL particles. Corroborating this view, T-0681 significantly reduced the net cholesterol efflux from macrophages to plasma of apoE KOs drawn at 4 weeks. In contrast, T-0681 treated macrophages incubated with serum from the 8-weeks study did not show alterations of net cholesterol efflux. Although the induction of macrophage SR-BI together with an obviously unfavorable lipid composition of lipoproteins may in part explain the divergent effects of T-0681 on atherosclerosis development observed at different time-points, further studies are necessary to understand which mechanisms are responsible for the induction of the hepatobiliary sterol metabolism occurring between 4 and 8 weeks of treatment, making thyromimetics either pro- or anti-atherogenic.

Recently it was shown that functional reverse cholesterol transport critically depends on biliary sterol secretion [24]. Thus, induction of ABCG5/G8 and CYP7A1 may well have positively influenced the RCT from macrophages to feces in our studies, which was significantly promoted by T-0681. Interestingly, in CETP Tg mice T-0681 increased hepatic SR-BI and LDLr, but had no effect on hepatic ABCG5/G8 and CYP7A1. Moreover, plasma CETP mass was decreased. Although there is functional receptor-mediated cholesterol uptake in livers of CETP Tg mice, the moderate decrease of plasma [^3^H]-cholesterol by T-0681 in the RCT study did not reach statistical significance, and fecal output of [^3^H]-sterols was found unaffected upon treatment. Previously, Rader and coworkers clearly showed that in CETP Tg mice a considerable amount of macrophage-derived [^3^H]-cholesterol is transferred from HDL to apoB-containing lipoproteins by CETP and subsequently cleared by the hepatic LDLr [25]. Taken together, both decreased plasma CETP mass as well as the unaffected biliary sterol metabolism may have impaired the RCT mechanism in T-0681 treated CETP Tg mice. Interestingly, in our previous study in rabbits, we found no influence of the employed thyromimetic on plasma CETP activity; thus, first data on CETP function from the on-going clinical trials with thyromimetics are heavily awaited, since impairment of CETP activity may be deleterious in humans.

T-0681 treatment leads to increased LDLr expression in livers of CETP Tg mice, in NZW rabbits which naturally express CETP in plasma [10], and also in SR-BI KO mice, but not in livers of WT mice or apoE KO mice on Western-type diet. It seems that hepatic upregulation of LDLr by thyromimetics occurs when SR-BI-mediated uptake of plasma cholesterol is reduced and/or abolished, either through plasma CETP activity or due to the lack of SR-BI.

In summary, the present study confirms the atheroprotective properties of a prolonged treatment with T-0681. Our data suggest that this pharmacological effect derives at least in part from the modulation of hepatic ABCG5/G8 and CYP7A1 expression. This study also suggests that LDLr expression is necessary for the lipid-lowering activity of selective thyromimetics. However, further studies in LDLr KO and ABCG5 KO mice would be helpful to clarify the specific role of the LDLr and of biliary sterol secretion in the prevention of atherosclerosis conferred by T-0681.

## Materials and Methods

### Reagents

The liver-selective thyromimetic T-0681 (former KAT-681) [26] was kindly provided by Kissei Pharmaceutical Co., LTD, Nagano, Japan.

### Studies in WT, SR-BI KO, and LDLr KO Mice

All animals were handled in strict accordance with good animal practice as defined by the Austrian Authorities, and all animal work was approved by the Austrian Animal Care and Use Committee. Male C57/B6 (WT) mice, obtained from Charles River Laboratories (Kisslegg, Germany), were fed a standard chow diet (Ssniff, Soest, Germany). After 2 weeks of acclimatization, mice were divided into two groups and subcutaneously implanted with Alzet micro-osmotic pumps (model 1002, Durect Corporation, Cupertino, CA, USA) carrying T-0681 in PBS (36 nmol/kg/d) or PBS alone as control for 14 days. After 14 days of treatment, animals were fasted for 5 h and anesthetized. Blood samples were taken, mice sacrificed by cervical dislocation, and organ biopsies were snap-frozen.

Male SR-BI KO and LDLr KO mice, obtained from Jackson Laboratories (Bar Harbor, Maine, USA) were fed a standard chow diet (Ssniff, Soest, Germany), and subcutaneously implanted with Alzet micro-osmotic pumps (model 1002, Durect Corporation, Cupertino, CA, USA) carrying T-0681 in PBS (36 nmol/kg/d) or PBS alone as control. Mice were then fasted for 5 h, blood samples were taken, mice were sacrificed by cervical dislocation, and liver biopsies were snap-frozen.

### Macrophage *In Vivo* RCT Studies in WT and CETP Tg Mice

J774 macrophages were obtained from the American Type Culture Collection (ATCC, Manassas, VA, USA) and cultured in supplemented DMEM medium (Invitrogen GIBCO, Carlsbad, CA, USA) at 37°C and 5% CO_2_. Macrophage *in vivo* RCT studies were performed as described [15], [16], [27]. In brief, male WT were systemically treated with T-0681 (36 nmol/kg/d) or PBS for 14 days using Alzet micro-osmotic pumps as described above; the treatment was continued during the 48-h RCT study. J774 macrophages were grown in suspension using a CELLspin 500 (Integra Biosciences, Chur, Switzerland) and radiolabeled with 2.5 µCi/ml [^3^H]-cholesterol (PerkinElmer, Boston, MA, USA) and 40 µg/ml acetylated LDL (AcLDL) [28] for 48 h. These foam cells were washed twice, equilibrated in medium with 0.2% bovine serum albumin for 6 h, spun down, and resuspended in PBS. The cholesterol content of J774 foam cells was markedly elevated, and the majority of cellular cholesterol was esterified (>80%), as determined by thin-layer chromatography. On day 12, [^3^H]-cholesterol–labeled and AcLDL-loaded J774 cells (typically 8×10^6^ cells containing 2×10^6^ counts per minute [cpm] in 0.6 ml PBS) were injected intraperitoneally. Feces were collected continuously from 0 to 48 h and stored at 4°C before extraction of cholesterol and bile acids. At study termination (48 h after injection), mice were exsanguinated, perfused with cold PBS, and portions of the liver were removed and snap-frozen. Liver lipid, fecal cholesterol as well as bile acid extractions were performed as described [27]. Radioactivity in plasma, liver as well as fecal lipid extracts was measured in a liquid scintillation counter. Macrophage in vivo RCT studies in male CETP Tg mice [29] on regular chow diet were carried out as described using 1×10^6^ J774 macrophages containing 0.65×10^6^ cpm in 0.6 ml PBS.

Macrophage in vivo RCT studies in male WT mice with primary murine bone marrow-derived macrophages (1×10^6^ cells containing 0.6×10^6^ cpm in 0.6 ml PBS) were performed as described elsewhere [30].

### Cholesterol Efflux Studies

J774 macrophages were labeled with [^3^H]-cholesterol (5 µCi/ml [^3^H]-cholesterol, PerkinElmer; 5 mg/ml ACAT inhibitor, Sigma) for 48 h. Then cells were washed and equilibrated overnight in either the presence or absence of T-0681 (100 nM) in serum-free medium. For the cholesterol efflux, medium containing 2.5% mouse plasma from the 4-weeks and the 8-weeks studies was added to cells. After 4 hours, supernatant was collected and centrifuged (5 min, 3000×g, 4°C). Cells were washed with PBS and lyzed in 1 ml 0.1 N NaOH. 300 µl of supernatant and cell lysate were measured in a liquid scintillation counter. Percentages of cholesterol efflux were calculated using following formula: 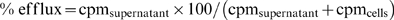


### Atherosclerosis Studies in apoE KO Mice

Male apoE KO mice were obtained from Jackson Laboratories (Bar Harbor, Maine, USA) After 2 weeks of acclimatization, mice were set on a Western type diet (Ssniff, Soest, Germany), divided into two groups and subcutaneously implanted with Alzet micro-osmotic pumps (model 1004, Durect Corporation, Cupertino, CA, USA) carrying T-0681 in PBS (36 nmol/kg/d) or PBS alone as control. After 4 and 8 weeks, mice were fasted for 5 h, blood samples were taken, mice sacrificed by cervical dislocation, and liver biopsies were snap-frozen. Hearts were prepared and atherosclerotic lesions quantified as described [31]. In brief, after sacrifice by cervical dislocation, the heart of each animal was perfused with 100 ml of PBS and fixed with 100 ml 4% phosphate-buffered paraformaldehyde (pH 7.0) and serial 10-µm-thick sections were cut between the valves and the aortic arch for quantitative analysis of lipid deposition. On average, 180–200 slices were collected per mouse. For histological analyses, every sixth slice was stained with Oil red-O and hematoxylin to identify atheromatous lesions. Images were captured by use of a JVC 3-charge–coupled device video camera. Sections were analyzed using the computer-assisted Quips Image analysis system (Leica Mikroskopic and System GmbH, Wetzlar, Germany).

### Lipid Parameters

Total cholesterol was measured in whole plasma of each animal employing an ABX Diagnostics commercial kit (ABX Diagnostics, Montpellier, France). Additionally, pooled plasma of each group was subjected to FPLC fractionation analysis with two tandem Superose 6 columns (GE Healthcare, Vienna, Austria) as described previously [32]. Apolipoprotein measurements were performed by an immunonephelometric assay as described [33]. Hepatic cholesterol content was measured as described [34]. CETP mass was determined using a previously developed ELISA [35].

### Fecal Sterol Analysis

50 mg of dried feces were boiled in 1 ml alkaline methanol (1M NaOH/Methanol, 1∶3 v/v) at 80°C for 2 h after addition of 50 nmol 5α-Cholestane as internal standard for neutral sterol analysis. After cooling down to room temperature, neutral sterols were extracted using three times 3 ml of petroleum ether, boiling range 60–80°C. The residual sample was diluted 1∶9 with distilled water. 100 µl of the solution were subjected to an enzymatic total bile acid measurement [36]. The extracted neutral sterols were converted to trimethylsilyl derivatives. Neutral sterol composition of prepared feces samples was determined by capillary gas chromatography on an Agilent gas chromatograph (HP 6890, Hewlett Packard, Palo Alto, CA, USA) equipped with a 25 m×0.25 mm CP-Sil-19 fused silica column (Varian, Middelburg, The Netherlands) and a Flame Ionization Detector. The working conditions were the following: Injector temperature 280°C; pressure 16.0 psi; column flow constant at 0.8 ml/min; oven temperature program: 240°C (4 min), 10°C/min to 280°C (27 min); detector temperature 300°C.

### Plant Sterol Measurement in Plasma

Sitosterol and campesterol were extracted from 10 µl plasma from each animal in duplicate samples. Samples were derivatized with trimethylsilane reagent (pyridin∶hexametyldisilan∶trimetylchlorosilane 3∶2∶1, v/v/v) prior to gas-chromatography-mass spectrometry analysis [37]. D5-campesterol/sitosterol was used as internal standard. The levels of sitosterol and campesterol in plasma reflect cholesterol absorption [12].

### Protein Extraction and Western Blot Analysis

Preparation of proteins from murine liver samples as well as from T-0681-treated J774 macrophages and subsequent Western blot analysis were performed as described [32]. Murine SR-BI was detected using NB 400-104 (Novus Biologicals, Littleton, CO, USA), ABCA1 was tested with a polyclonal rabbit anti-ABCA1 antibody (NB 400–105; Novus Biologicals). Anti-LDLr antibody was a generous gift from J. Herz [38]. The chemoluminescent reaction was performed using Super Signal West Dura Reagent (Pierce, Rockford, IL, USA) and blots were visualized by Fluor-S-Imager using Quantity One V4.1 software (BioRad, Hercules, CA, USA).

### RNA Isolation, Reverse Transcription, and Quantitative Real-Time PCR

Total RNA was extracted using RNA bee according to the manufacturer's protocol (Tel-test Inc., Friendswood, Texas, USA) and reverse transcribed with Omniscript-RT Kit (Qiagen, Hilden, Germany). Primers and probes for murine ABCA1, ABCG5, ABCG8, CYP7A1 were described previously [27], primers and probes for murine NPC1L1 elsewhere [39]. GUSB and HPRT were used as reference (Applied Biosystems, Foster City, CA, USA). Taqman real-time PCR reactions were performed on a Mx4000® Multiplex Quantitative PCR System (Stratagene, Amsterdam, The Netherlands).

### Statistics

Results are presented as mean ± s.e.m. The statistical significance of the differences between the means of the experimental groups was tested by the Student's *t*-test for unpaired data. A difference was considered statistically significant when *P*<0.05.
